# The influence of hallux valgus severity on postural stability and risk of fall: a cross-sectional study design

**DOI:** 10.1186/s12891-025-09357-6

**Published:** 2025-12-11

**Authors:** Kholoud M. Omar, Alaaeldin A. K. Mohamed, Mohammed M. M. Essa, Mahmoud Y. Elzanaty, Marwa M. Mahfouz Mahmoud

**Affiliations:** 1 Demonstrator at The Basic Science Department, Faculty of Physical Therapy, Deraya University, Minya, Egypt; 2 Department of Physical Therapy, Benha University Hospital, Benha University, Benha, Qalyubia Egypt; 3 Orthopedic Physical Therapy Department, Orthopedic Physical Therapy, Deraya University, Minya, Egypt; 4 Associate professor of Biomechanics, Faculty of physical therapy, Sphinx University, Assuit, Egypt; 5 Physical Therapy for Neurology and Neurosurgery disorder, Faculty of physical therapy, Cairo University, Giza, Egypt; 6 Physical Therapy for Neurology and Neurosurgery disorder, Faculty of physical therapy, Deraya University, Minya, Egypt; 7 Associate professor at The Basic Science Department, Physical Therapy, Deraya University, Minya, Egypt

**Keywords:** Hallux valgus, Biodex-balance system, Postural stability, Risk of fall, Visual analogue scale (VAS), Manchester scale

## Abstract

**Background:**

Hallux valgus (HV) is a common first-ray foot disease that causes changes in joint mechanics, abnormal loading patterns, and structural imbalances within the foot, leading to pain, functional impairment, and changes in gait and posture.

**Purpose:**

The current study was designed to examine postural stability, risk of falls, and pain in adults with mild, moderate, and severe hallux valgus and to compare the results among these groups.

**Method:**

Eighty female subjects took-part in this study. They were selected from the Deraya University, with ages ranging from 20 to 40 years. All procedures were clearly explained to each participant before beginning the study. The subjects were distributed into four equal groups (20 subjects each) based on the severity of hallux valgus (HV): Group A: control group, Group B: Mild HV, Group C: Moderate HV, Group D: Severe HV. All subjects were assessed using the Visual Analogue Scale (VAS), the Manchester Scale, a Universal Goniometer, and the Biodex Balance System. These tools were employed to evaluate pain intensity, severity of the deformity, postural stability, and risk of fall.

**Result:**

No significance difference was detected among groups concerning age, weight, height, Body mass index (*p* >0.05). A significant increase in antro-posterior stability index, mediolateral stability index, static and dynamic overall stability index, sway velocity index open eyes, sway velocity index closed eyes and VAS was observed in severe HV compared with control subjects and other study groups (*p* < 0.001).

**Conclusion:**

Hallux valgus has a detrimental impact on postural stability and should be recognized as a significant as well as independent risk factor for falls. In addition to balance impairment, hallux valgus is also associated with increased foot pain.

## Background

The human foot is a complex anatomical structure that plays a vital role in lower extremity biomechanics. In addition to providing shock absorption, weight-bearing support, and enabling locomotion, the foot offers a stable base and is capable of withstanding deformation. As the most distal part of the lower limb and the first to contact the ground during gait, the foot is essential for maintaining balance and efficient movement [1, 2].

The ability to maintain stable standing posture is essential for many daily activities. Although postural balance relies on the coordinated function of the sensory, neurological, and motor systems, age-related and pathological changes in these systems can impair standing balance and increase the risk of falls. Additionally, foot-related problems are recognized as significant contributors to impaired postural control [3].

The foot helps maintain stability in two main ways: (i) by giving the body mechanical support through the arch’s osteoligamentous architecture and the coordinated action of lower limb muscles, and (ii) by using plantar tactile mechanoreceptors to provide sensory information about body position. This support function is therefore probably compromised by abnormalities in foot posture, flexibility, strength, or sensitivity, which can lead to loss of balance [4].

Hallux valgus (HV) is considered a prevalent deformity of the 1 st ray of the foot. Dysfunction of meta-tarsophalengeal (MTP) joint contributes to pain, deformity, and functional limitations, often affecting patients’ daily activities. HV is a frequently encountered condition in traumatology and orthopedics and is reported to occur up to ten times more often in women than in men [5]. Additionally, its prevalence has been found to be approximately twice as high in white populations compared to Black African populations [6].

The severity of HV is commonly evaluated through a combination of clinical and radiographic methods to enhance diagnostic accuracy and consistency. Clinically, tools such as the Manchester Scale offer a standardized visual grading system based on photographic representations of increasing deformity severity.In addition to visual grading, the hallux valgus angle (HVA) can be measured using a Universal goniometer during weight-bearing to provide a quantitative assessment. Radiographic evaluation, particularly with weight-bearing dorsoplantar (DP) foot X-rays, remains the gold standard for diagnosing HV, as it enables precise anatomical measurement of the HVA Based on radiographic assessment, HV severity is typically classified as follows: Mild HV: HVA < 20°,Moderate HV: HVA between 20° and 40°and Severe HV: HVA >40° [7].

The distribution of plantar pressure is changed by an abnormal HVA, which usually causes a medial shift in foot loading towards the 2nd and 3rd metatarsal heads. These biomechanical changes, coupled with muscle imbalances, can disrupt normal foot function and compromise postural stability [8]. Additionally, HV has also been strongly linked to an increased risk of falls [9, 10]. Furthermore, some studies have found that people with HV experience a decline in overall well-being and daily functioning. According to these studies, HV may affect an individual’s general health and life style in addition to causing isolated foot issues [11].

Therefore, further investigation into the relationship between hallux valgus and balance impairment is needed to provide a more comprehensive understanding that may inform effective prevention and rehabilitation strategies aimed at improving balance and reducing the risk of falls. Although hallux valgus is more common in older adults, the present study focused on young adult women (20–40 years) to minimize the confounding influence of age-related decline in postural control and neuromuscular function, allowing a clearer examination of the independent impact of hallux valgus deformity. Accordingly, the study’s objectives were to assess postural stability, fall risk, and pain among individuals with mild, moderate, as well as severe HV and compare the outcomes between these groups.

## Methods

### Study design

This cross-sectional study aimed to examine postural stability, risk of falls, and pain in adults with mild, moderate, and severe hallux valgus and to compare the results among these groups. It was carried-out at the Faculty of Physical Therapy, Deraya University, El-Minya, Egypt, from 8 August to 25December 2024.

### Participants

Eighty female participants were enrolled, including sixty with hallux valgus and twenty control participants. A subject was included in the study if their age ranged from 20 to 40 years, their body mass index (BMI) ranged from 18.5 to 24.9 kg/m^2^, their big toe’s MTPJ was painful. Subjects were excluded if they had lower extremity malalignment, inflammatory arthritis, neurological, cognitive, mental, psychological disorders, received any non-operative treatment for HV in the last 6 months, and falling phobia.

### Instruments

A. The Visual Analogue Scale (VAS), a single-dimension for measurement of pain intensity, was one of the most widely utilized tools for assessing pain in clinical and research settings. It was a simple effective instrument that allows patients to subjectively rate their pain intensity along a continuum, typically represented by a 10-centimeter line. Numerous studies have confirmed its validity and reliability, with research by [12] as well as [13] supporting its moderate to good reliability for evaluating pain.
B. Manchester Scale, a scaling system that divides the hallux angle into levels of severity, instead of measuring the exact angle, was the easiest and most widely stated method by healthcare providers and researchers. Garrow and colleagues first developed the Manchester Scale in 2001. It does serve as reliable classification of severity [14]. It has been proven to be valid in comparison to radiographic evaluations [15]. When precise hallux angle is not necessary, the Manchester Scale is generally considered a valid replacement for radiographic evaluation [16].
C. Universal Goniometer was a crucial evaluation skill in musculoskeletal medicine, and the resulting measurements were utilized to guide treatment actions and ascertain whether dysfunction existed and generate evidence of treatment effectiveness. Universal Goniometer demonstrated good overall intra- and interQ7 2 tester reliability, and a sufficiently valid measurement tool when compared to radiography. The universal goniometer involved two overlying clear plastic arms connected by a 360° protractor at the center, with a usual total length of 30.5 cm. A degree-graduated protractor is attached to the stationary arm, and a reference line traverses the middle and follows the whole length of the moving arm. As the movable arm rotates relative to the fixed arm, the reference line sweeps across the graduated scale, indicating the precise angle of movement [17, 18].
D. Biodex Balance System (BBS), the subject’s postural stability in addition to risk of fall were examined utilizing the BBS. This system features a high-resolution color touch-screen LCD display, an integrated color printer with a separate stand, and the capability to perform both static and dynamic balance assessments [19]. The validity and reliability of the Biodex balancing system as a tool for evaluating static and dynamic stability as well as fall risk have been established. Both the high intra-class correlation (ICC) and the proportion of variation in method error were low in the FRI, indicating high (ICC). According to the ICC, the Overall Stability Index (OSI) demonstrated an acceptable level of reliability [20].

### Procedures

**Fig. 1:**
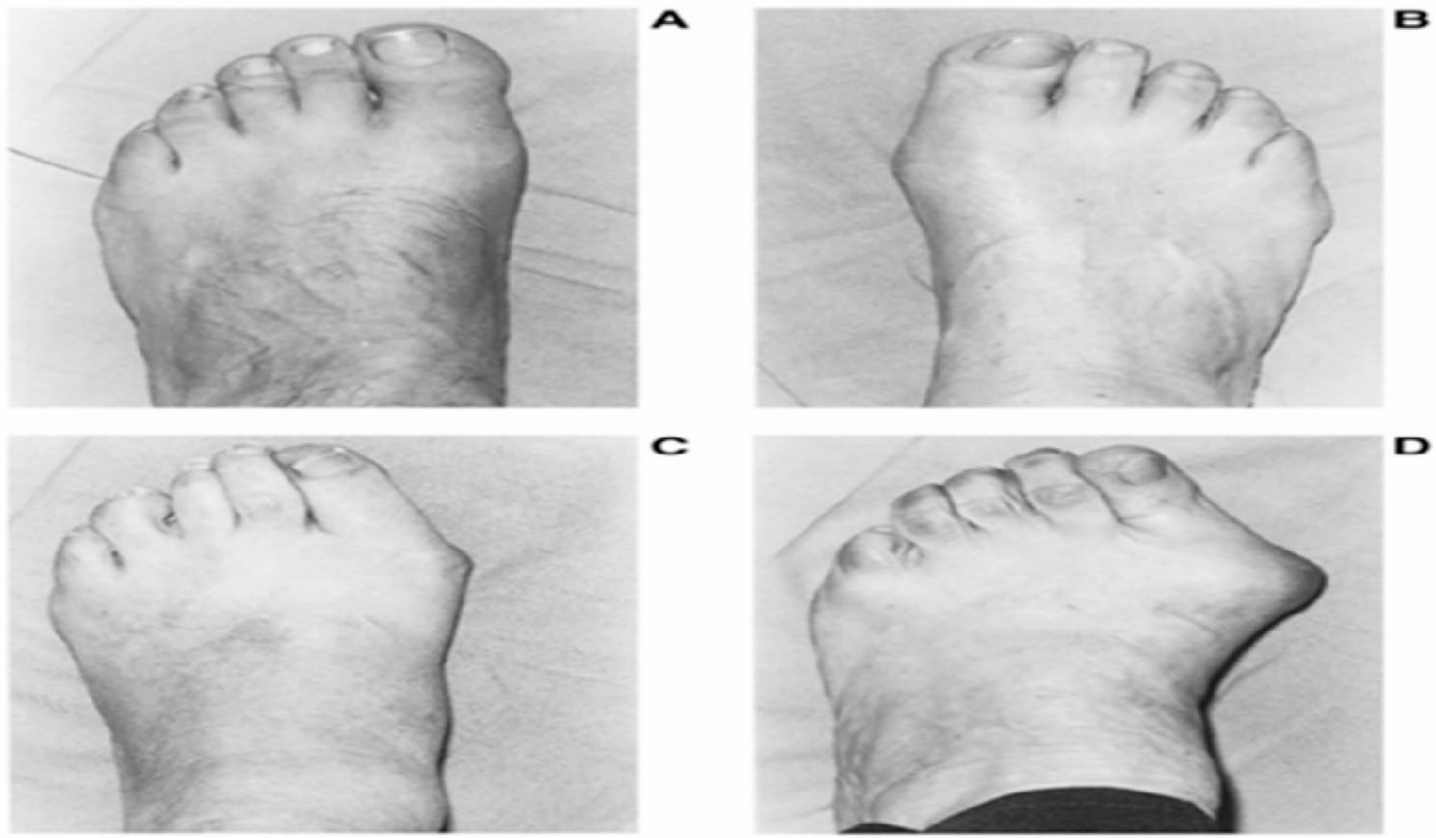
Manchester scale
  B. Universal Goniometer: The angle formed by the longitudinal axes of the proximal phalanx as well as the 1 st metatarsal is known as the HV angle, was measured using a goniometer from the dorsal aspect of the foot while participants stood in a relaxed, weight-bearing position. The goniometer’s axis was positioned over the 1 st MTP joint, with the fixed arm lined up with the1st metatarsal’s shaft while the moving arm was aligned up with the hallux’s longitudinal axis. The HVA was recorded in degrees. An HVA of 15° or less was considered normal; HV was considered mild when the angle was less than 20°, moderate when it was between 20° and 40°, and severe when it was greater than 40° (Fig. 2) [22].

A. Pain assessment: The intensity of foot pain was assessed using (VAS). Subjects were asked to report pain in the first (MTP) joint while standing in a weight-bearing position on both feet. On a 10-centimeter visual analog scale (VAS), from 0 (no pain) to 10 (the most severe pain possible), each participant determined the intensity of their pain [21].
B. Hallux valgus severity
  A. Manchester scale: The Manchester Scale categorizes hallux valgus deformity into four grades based on standardized clinical photographs illustrating progressive increases in the hallux valgus angle. The severity of HV in this study was assessed using this scale, which was administered by a trained expert under standardized conditions. The classification includes: Grade 1 (A) – no deformity, Grade 2 (B) – mild deformity, Grade 3 (C) – moderate deformity, and Grade 4 (D) – severe deformity Fig. (1).

**Fig. 2:**
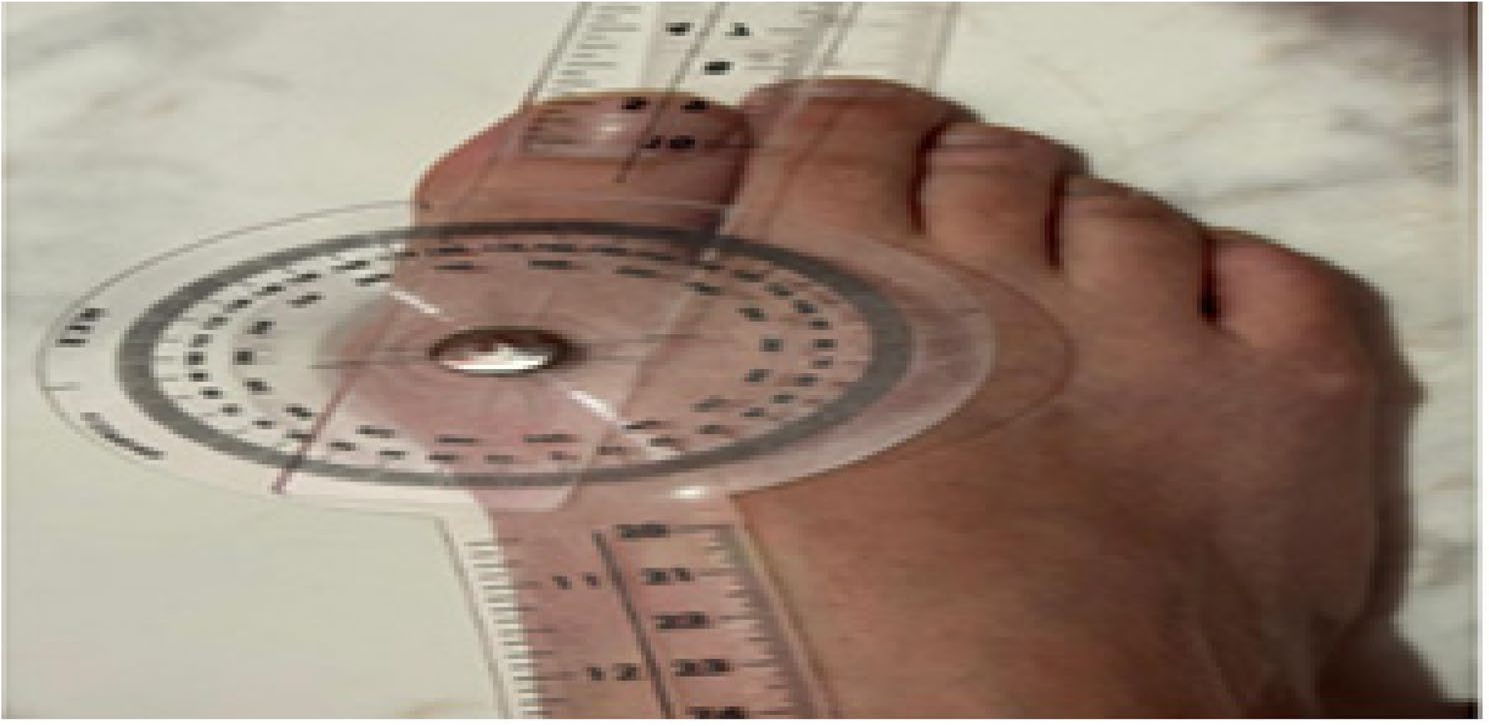
Measurement of hallux valgus angle from dorsal aspect of the foot while participants stood in a relaxed, weight-bearing position by Universal Goniometer
  C. Measurement of postural stability and risk of fall utilizing biodex balance system (BBS): To evaluate postural stability as well as risk of fall, the Biodex Balance System (Model: TP-3040-15, Made in Taiwan) was used. The platform of the device provides a resistance of movement (stability levels of the platform) from 1 (most stable) to 12 (least stable).

The participants were tested for:

i. Anterior/Posterior stability index (APSI).
ii. Medial/Lateral stability index (MLSI).
iii. Overall stability index (OSI).
iv. Sway velocity index (SVI).

For the assessment of static postural stability, the testing parameters were set to provide 30-second trials with the platform steadiness fixed at level 1. For dynamic postural stability testing, the same as static PS, but the platform steadiness was varied across levels 2 to 8. Each subject completed one training trial followed by three testing trials, with the subjects s’ eyes open, using a ten-second rest period among each trial. The mean score from the three testing trials was then taken for analysis [23]. Subjects were asked to stand in a natural, relaxed stance on the static platform of the (BBS). The “Position Patient” screen displayed the subject’s center of gravity as a dot, and foot placement was adjusted until the dot was positioned on or near the central axis of the screen. Once properly aligned, the subject’s heel position and foot angle were recorded based on the platform’s grid coordinates and entered into the system. Subjects were then instructed to maintain their center of gravity within the smallest concentric rings (balance zones) displayed on the BBS monitor. To ensure consistency during testing, subjects were told not to move their feet and to keep the platform level, stable, and as immobile as possible. They were asked to stand upright, gaze forward, and relax their arms at their sides throughout the trial period [24].

In order to measure risk of fall, the subjects were submitted to the fall risk protocol of BBS and asked to step on the platform bilaterally without footwear at all times of measurements looking forward with the focusing of maintaining upright relaxed position. The test measures the subject postural sway velocity to predict risk. Then, the measurements were applied one time in eyes open and another in closed position with the participant in a narrow stance for 45 s for each test with (30-s) rest in between [25].

### Statistical analysis

The number of participants needed for this study was determined to be *N* = 76 utilizing the G*POWER statistical program (version 3.1.9.2; Franz Faul, Universitat Kiel, Germany) in conjunction with the postural sway data obtained from [26]. The effect size is 0.4, power is 80%, and α is set at 0.05 for the calculation.

The analysis of variance (ANOVA) test was used to compare the subjects’ characteristics across groups. To ensure that the data followed a normal distribution, the Shapiro-Wilk test was used. The homogeneity of variances among groups was evaluated using Levene’s test. We compared the groups using APSI, MLSI, static and dynamic OSI, SVIOE, SVICE, and VAS using a one-way ANOVA. The Tukey test for multiple comparisons was used to do post hoc comparisons. A *p*-value less than 0.05 was used to determine statistical significance for all tests. We used SPSS 25 for Windows (IBM SPSS, Chicago, IL, USA) to complete the statistical analyses.

## Results

### Subject characteristics

Table 1 shows the characteristics of the individuals. Individuals with mild, moderate, or severe HV did not differ significantly from controls with respect to age, weight, height, or BMI (*p* > 0.05).

**Table 1:** Subject characteristics

	control subjects	Mild HV	Moderate HV	Severe HV	F- value	*p*-value
	Mean ± SD	Mean ± SD	Mean ± SD	Mean ± SD		
Age (years)	32.45 ± 2.72	31.15 ± 3.15	31.20 ± 3.38	32.60 ± 3.30	1.23	0.3
Weight (kg)	62.75 ± 6.16	61.80 ± 9.43	62.15 ± 6.70	62.20 ± 5.77	0.06	0.98
Height (cm)	162.55 ± 5.92	162.20 ± 5.14	163.05 ± 5.48	161.45 ± 5.21	0.31	0.82
BMI (kg/m²)	23.80 ± 2.59	23.40 ± 2.43	23.39 ± 2.39	23.87 ± 2.05	0.22	0.87

*SD* Standard deviation, *MD *Mean difference, *p* value, Probability value

### Impact of HV severity on postural stability and sway velocity

A significant difference was observed in APSI, MLSI, static along with dynamic OSI, SVIOE, and SVICE between control subjects and those with HV (*p* < 0.001).

The results showed that APSI, MLSI, static and dynamic OSI, SVIOE, and SVICE were significantly higher in subjects with severe HV as compared to control individuals and those with mild or moderate HV (*p* < 0.05). But comparing those with moderate and mild HV, no statistically significant difference was found (*p* > 0.05).

In comparison to control subjects, individuals with moderate HV showed a significant increase in static OSI, SVIOE, as well as SVICE (*p* < 0.05). In terms of APSI, MLSI, and dynamic OSI, however, neither control subjects nor those with mild or moderate HV differed significantly (*P* > 0.05).

A significant increase in VAS in subjects with s severe HV compared with mild and moderate HV; and a significant increase in moderate HV compared to mild HV (*p* < 0.05). (Tables 2 and 3).

**Table 2:** Mean APSI, MLSI, static and dynamic OSI, SVIOE and SVICE, of control subjects and subjects with mild, moderate and severe HV

	Control subjects	Mild HV	Moderate HV	Severe HV	F- value	*p*-value
	Mean ± SD	Mean ± SD	Mean ± SD	Mean ± SD		
APSI	1.36 ± 0.29	1.39 ± 0.30	1.41 ± 0.34	3.62 ± 0.39	229.55	0.001
MLSI	0.58 ± 0.28	0.61 ± 0.27	0.68 ± 0.24	1.15 ± 0.29	19.46	0.001
Static OSI	0.41 ± 0.18	0.53 ± 0.19	0.59 ± 0.21	1.31 ± 0.24	76.29	0.001
Dynamic OSI	1.53 ± 0.25	1.60 ± 0.23	1.62 ± 0.29	4.63 ± 0.28	658.87	0.001
SVIOE (mm/s)	11.85 ± 1.44	12.44 ± 1.78	13.41 ± 1.51	14.82 ± 1.92	12.01	0.001
SVICE (mm/s)	13.96 ± 1.71	15.34 ± 2.24	15.88 ± 1.91	18.12 ± 2.20	14.58	0.001
VAS	-	2.15 ± 1.27	4.20 ± 1.47	6.45 ± 1.64	42.98	0.001

*SD* Standard deviation, *p* value, Probability value

**Table 3:** Comparison between control subjects and subjects with mild, moderate and severe HV

Outcome	control vs. Mild HV		control vs. Moderate HV		control vs. Severe HV		Mild HV vs. Moderate HV		Mild HV vs. Severe HV		Moderate HV vs. Severe HV	
	MD	*p* value	MD	*p* value	MD	*p* value	MD	*p* value	MD	*p* value	MD	*p* value
APSI	−0.03	0.99	−0.05	0.95	−2.26	0.001	−0.02	0.99	−2.23	0.001	−2.21	0.001
MLSI	−0.03	0.99	−0.1	0.65	−0.57	0.001	−0.07	0.85	−0.54	0.001	−0.47	0.001
Static OSI	−0.12	0.31	−0.18	0.03	−0.9	0.001	−0.06	0.75	−0.78	0.001	−0.72	0.001
Dynamic OSI	−0.07	0.84	−0.09	0.71	−3.1	0.001	−0.02	0.99	−3.03	0.001	−3.01	0.001
SVIOE (mm/s)	−0.59	0.68	−1.56	0.02	−2.97	0.001	−0.97	0.26	−2.38	0.001	−1.41	0.04
SVICE (mm/s)	−0.59	0.68	−1.56	0.02	−2.97	0.001	−0.97	0.26	−2.38	0.001	−1.41	0.04
SVICE (mm/s)	−1.38	0.15	−1.92	0.01	−4.16	0.001	−0.54	0.83	−2.78	0.001	−2.24	0.004
VAS	—	—	—	—	—	—	−2.05	0.001	−4.3	0.001	−2.25	0.001

*SD* standard deviation, *MD *mean diff

## Discussion

This study was done to examine the influence of HV severity on postural stability, risk of fall as well as pain. Sixty female subjects with mild, moderate, in addition to severe HV participated in this study and were compared with control subjects. Data obtained from the study groups regarding (APSI), (MLS), Overall Stability Index (OSI), (SVI) and (VAS) were statistically analyzed and compared. A frequent foot deformity known as HV involves the gradual lateral displacement of the hallux, subluxation of the 1 st MTP joint, and an occurrence of osteoarthritis. Overall health and everyday functioning are significantly affected by increasing HV severity, and HV is associated with an increased risk of falls, particularly in older persons [26].

### Regarding the comparison of static and dynamic postural stability index (SI) between control subjects and subjects with mild, moderate along with severe HV

The current study’s findings align with those of Menz and Lord [27], who reported a significant correlation between the degree of hallux valgus (HV) deformity and balance performance. This relationship can be explained biomechanically: the medial displacement of the first metatarsal and lateral deviation of the great toe may compromise the anatomical alignment of the forefoot, thereby disrupting load distribution and producing retrograde forces on the metatarsal head that impair stability [28].

Additionally, the present study’s findings are in agreement with those of a previous study suggesting that severe HV deformity contributes to gait alterations and increases the risk of falling, particularly in older adults [28, 29]. Individuals with moderate to severe HV exhibit greater instability when walking on uneven or challenging surfaces, likely due to the combined effects of mechanical imbalance, altered proprioception, and reduced efficiency of postural control mechanisms, which together lead to a pronounced impairment in both static and dynamic balance.

The severity of the HV was classified as mild, moderate or severe within a study by [30] for the patients. According to the present study, people with mild HV appeared to have normal postural sway, but those having moderate to severe HV had significantly shorter steps and lower velocities compared to the no HV or mild HV groups.

Muscle weakness surrounding the 1 st MTPJ, greater mediolateral postural sway, and severe foot-specific pain and impairment are all symptoms of HV deformity, according to [31].

This study’s findings are in line with those of a previous one that used the evaluation tools (the Timed Up and Go test, the Berg Balance Scale, as well as the Falls Efficacy Scale) to compare the effects of HV and non-HV on 20 female patients. Walking speed, balance, and postural stability were all negatively impacted in the HV group, and they were also had high risk of falling.

This study’s results contradict those of a previous one that found that mild HV angles had a deleterious effect on single-limb balance [32]. Mediolateral stability scores demonstrated higher stability ratings in individuals with pathological mild HV angles compared to normal feet, and participants with these angles had a reduced capacity to maintain stability.

This study’s findings contradict those of a previous one Kavlak [33], that found no correlation between hallux valgus anterior (HVA) and either static or dynamic balance outcomes, suggesting that the severity of HV doesn’t indicate balance but does influence the functional capacity of the forefoot in older men to some extent.

### Regarding on Sway Velocity Index even with Open Eyes (SVIOE) or Closed Eyes (SVICE) between control subjects and subjects with mild, moderate and severe HV

These results are in agreement with those of Liu et al. [34], who found that the calf muscles of older people with HV are underactive at the joints of the foot; furthermore, even when the thigh muscles compensate, the individual still has an imbalance and is high risk of falling. The findings of this study corroborate those of previous research in the elderly, which has shown decreased lateral stability, impaired coordinated stability, and greater postural sway, all of which are thought to be independent risk factors for falls [15, 35].

Also, current study was in consistent with the study done by Sánchez-Sanjuan et al. [36] who came to the conclusion that changes in foot morphology, like as those seen in HV, impact stability and thereby heighten the risk of falls among the elderly. This relationship underscores the biomechanical importance of proper foot alignment in maintaining balance, as deformities in the first ray can disrupt the medial longitudinal arch, alter plantar pressure distribution, and reduce the efficiency of postural adjustments required for stability. In line with this, the risk of falls and pathology increases with age and with being female. In addition, a cohort study conducted by Menz and Morris [37],, found that HV was the reason behind falls in women over the age of 70, and those who fell had more severe HV deformity compared to those who didn’t fall, suggesting that progressive structural and neuromuscular alterations in the forefoot may compromise postural control mechanisms and heighten fall susceptibility.

In terms of biomechanics, the hallux and the 1 st MTP joint are crucial for stability and gait. The hallux impacts the propulsive phase in walking. Behaving as a pivot, the first metatarsophalangeal joint facilitates the forward transference of the body [38]. One possible consequence of diminished great toe force generation capacity is a potential slowing of peak swing speed in elderly patients with HV [39, 40].

### Pain intensity (VAS) and its association with hallux valgus severity

In relation to the VAS, the present study found a statistically significant difference in VAS measures between the control group and the groups with mild, moderate, as well as severe HV.Normally, by increasing the severity the level of pain particularly in the first (MTPJ) would increase during ADLS and during day time while using fitting shoes, also pain might associate to decrease muscle strength around the muscles of the foot.

This study’s findings are in line with those of a previous one that found that HV increased the prevalence of self-reported foot pain and decreased the quality of self-reported physical functioning in individuals aged 40 and up [41]. Researchers also noted that those aged 30 and up (*n* = 3082) with HV alone, big toe pain only, or HV plus big toe pain had a worse health-related quality of life, thus emphasizing the significance of big toe pain in conjunction with HV. Muscle weakness surrounding the1st MTPJ, heightened mediolateral postural sway, and severe foot-specific discomfort and impairment are all symptoms of HV deformity, according to [31].

Additionally, Jankowicz-Szymańska et al. [42], found that both the younger group of women (those under 60 years old) as well as the older group (those 60 years and up) who had HV felt foot pain and toe area pain. Pain in the foot along with big toe area was most commonly reported by women aged 60 and up, but younger women complained of similar amounts of pain. Finally, it was found that HV deformity was associated with pain can affect balance and risk of fall according to its severity as severe cases were the most prominent group.

This study’s findings contradict those of previous research conducted by Nix et al. [43], who reported no significant linear association between the hallux valgus angle and pain intensity measured by the Visual Analog Scale (VAS). Their results suggested that the degree of angular deformity alone may not adequately explain the variability in pain perception among individuals with hallux valgus.

Similarly, Hurn et al. [44] found that the hallux valgus angle did not significantly predict pain severity in multivariate analyses, indicating that other factors such as soft tissue inflammation, altered joint loading, or individual pain sensitivity might play a more dominant role in pain generation.

### Limitations

It is important to address a number of methodological concerns raised by the present study. The first limitation is that the study’s cross-sectional design makes it difficult to draw any firm conclusions about the causal relationship among HV and the measured outcomes of balance as well as fall risk. Additionally, the current study faced challenges in recruiting a required number of participants who met the study’s specific inclusion criteria, particularly individuals with severe hallux valgus within the 20–40 age range. Furthermore, some participants experienced apprehension or anxiety during the balance assessments, especially due to fear of fall.

## Conclusion

According to the results of this study, it can be concluded that HV has a detrimental impact on postural stability and should be recognized as a significant as well as independent risk factor for falls. HV is a musculoskeletal disorder commonly associated with neuromuscular control abnormalities, characterized by a marked lateral displacement of the great toe from the body’s midline; this deviation can lead to altered weight distribution, diminished balance, and unbalanced gait patterns. As a result, individuals with HV exhibit excessive postural sway throughout both static and dynamic activities compared to those without the deformity. Finally, the deformity was severe enough to cause further influences balance performance. Given its negative effects on gait and postural control, and it’s among the foot conditions most strongly associated with fall incidence. 

## Data Availability

The data that support the findings of this study are available from the corresponding author upon reasonable request.

## Abbreviations

HV: Hallux valgus
VAS: Visual analogue scale
MTP: Meta-tarsophalengeal
BMI: Body mass index
BBS: Biodex balance system
ICC: Intra-class correlation coefficient
OSI: Overall stability Index
APSI: Anterior/Posterior stability index
MLSI: Medial/Lateral stability index
SVI: Sway velocity index
SI: Stability index
SVIOE: Sway velocity index even with open eyes
SVICE: Sway velocity index even with closed eyes
